# Special Issue: Emerging Wildlife Viral Diseases

**DOI:** 10.3390/v14040807

**Published:** 2022-04-13

**Authors:** Subir Sarker

**Affiliations:** Department of Microbiology, Anatomy, Physiology and Pharmacology, School of Agriculture, Biomedicine and Environment, La Trobe University, Melbourne, VIC 3086, Australia; s.sarker@latrobe.edu.au; Tel.: +61-3-9479-2317

The past several decades have seen the emergences of novel viral infectious diseases increase steadily in wildlife populations globally [1,2,3,4,5,6,7,8]. Emerging viral diseases are acknowledged as an apparently growing trend of threats to wildlife and act as the source of a series of high-impact diseases recently emerging as pathogens affecting humans [9]. Most emerging viral pathogens, including Ebola and Marburg virus, human immunodeficiency virus virus-1 and -2, Nipah, Sin Nombre virus, Hendra and Menangle virus, West Nile virus, Middle East respiratory syndrome, and different subtypes of avian influenza, originate in wildlife and spill over into human hosts due to a range of ecological, demographic, and socio-economic changes [10]. Diseases caused by viruses, recently exemplified by the spread of SARS-CoV-2 (COVID-19) to human populations, also threaten wild animals from amphibians to mammals [8]. Habitat destructions, pollution, and international trade are among the factors contributing to a growing opportunity for viruses to spread to new hosts and cause disease.

Three independent studies deal with the emergency of novel viruses such as the protoparvovirus [11], a number of mongoose-associated circoviruses and cycloviruses [12], and a poxvirus, cheloniid poxvirus 1 (ChePV-1) [1]. Using various deployed approaches, the detected novel Newlavirus, a protoparvovirus of foxes in Newfoundland and Labrador, was shown to be 38.7–54.1% identical to the members of the genus *Protoparvovirus*, compared to NS1 proteins [11]. Although some aspects of novel protoparvovirus ecology were examined, future studies will require the assessment of the host specificity and its geographical distribution. In a study on *Circoviridae* [12], 76 of 83 of apparently healthy small Indian mongooses were investigated using PCR and determined complete genomes of three mongoose-associated circoviruses and six cycloviruses. Although the detected circoviruses in this study show a strong phylogenetic congruence with other animal and human origin circoviruses, it is unknown whether the detected circoviruses/cycloviruses have the ability to replicate in the mongoose host or are derived from dietary origin during prey–predator interaction [12]. 

Over the past several decades, multiple novel viruses have been found in wildlife species, including reptiles, and often pose a major threat to vulnerable species. Sea turtles are currently listed as vulnerable, endangered, critically endangered, or data deficient, depending on the species. In this Issue, a novel poxvirus, cheloniid poxvirus 1 (ChePV-1) [1], is characterized in a green sea turtle, associated with skin lesions, which is closely related to an avian poxvirus, demonstrating that interspecies transmission of potentially pathogenic viruses is occurring. This is the first comprehensive study that demonstrates evidence of poxvirus infection in a marine turtle species, as well as a rare example of an avipoxvirus crossing the avian-host barrier.

Rapid and sensitive diagnostic methods are crucial for the management of wildlife diseases and animal pandemic reservoir surveillance. In this Issue, two articles focus on the development of a quantitative polymerase chain reaction (RT-qPCR) for the rapid and accurate diagnosis of canine distemper virus [13] and leporid gammaherpesvirus 5 (LeHV-5) [14]. Both the developed methods were shown to be highly sensitive and specific for detecting individual viruses in the clinical samples. Moreover, it is paramount that we should continue to improve our veterinary and wildlife diagnostic capabilities to tackle existing and emerging viral diseases.

This Special Issue also covers two reviews that focus on the persistence of flavivirus infection in wildlife populations [15] and non-human vertebrates in Australia [16]. One focuses on the recent findings on flaviviruses and argues why we should continue the study on viral persistence, especially regarding flaviviruses, in wildlife populations [15]. In the absence of FDA-approved therapy, the elimination and control of spreading flavivirus infection largely relies on the host immune response. Therefore, we need a robust understanding of the pathophysiology of flavivirus infections, where cellular mechanisms trigger the establishment of persistent infection, for the development of therapeutic approaches. Another review highlights the complex nature of the mosquito-borne disease and the challenges of assessing the impacts on non-human vertebrate species in Australia [16]. Indeed, Australia houses several endemic mosquito-borne viruses, such as the Ross River virus, Barmah Forest virus, and Murray Valley encephalitis virus, which can significantly impact wildlife, livestock, and companion animals and could eventually cause significant changes to the Australian ecology and economy. 

This Special Issue has brought together contributions from multiple disciplines, including virology, wildlife disease diagnostics, and epidemiology, which will further strengthen our understanding of wildlife viruses, and notably, this will stimulate further the exploration of many important groups of wildlife viruses in the future. There remain many areas to be investigated, even in those very same areas dealt with in this Issue, including bringing attention to recurrent or new viral diseases affecting wildlife and discussing recent advances in diagnostics, pathogenesis, host-pathogen interactions, and evolutionary ecology. Finally, I would like to thank the authors who contributed to this Special Issue and the reviewers for their fruitful input.
